# Perceptions of Medical Students About Bedside Teaching in a Medical School

**Published:** 2018-06-30

**Authors:** Ajaya Kumar Dhakal, Devendra Shrestha, Suraj Bajracharya, Amita Pradhan, Balman Singh Karki, Sanjaya Dhakal

**Affiliations:** 1 Department of Pediatrics, KIST Medical College Teaching Hospital, Imadol, Lalitpur, Nepal; 2 Department of Orthopedics, KIST Medical College Teaching Hospital, Imadol, Lalitpur, Nepal; 3 Department of Community Medicine, KIST Medical College Teaching Hospital, Imadol, Lalitpur, Nepal; 4 Department of Microbiology, KIST Medical College Teaching Hospital, Imadol, Lalitpur, Nepal; 5 Department of Pediatrics, University of Alberta, Edmonton, Canada

**Keywords:** *bedside teaching*, *medical education*, *medical students*

## Abstract

**Introduction:**

Bedside teaching is an important and established learning tool in medical education. However there is a decline in bedside teachings over the years throughout the world including Nepal, due to advancement in medical technology, clinical skills labs and simulation techniques. This study aimed to find out the perception of Nepalese medical students towards different domains of bedside teaching.

**Methods:**

This was a descriptive cross-sectional study. A questionnaire consisting of Likert scale, open ended and closed ended questions was developed on different aspect of bedside teaching and the filled questionnaires were included for analysis.

**Results:**

Three hundred and six questionnaires were included. Almost all of medical students responded that bedside teaching is a useful learning modality in clinical teaching 304 (99.3%) and provides active learning in real context 291 (95%). The majority of medical students 233 (76%) were satisfied with the steps of history taking, examination followed by management discussion employed at bedside teaching. The students 223 (73%) were satisfied, how to elicit signs following demonstration of clinical exam by teachers at bedside. However majority 196 (64%) felt lack of individual opportunity at bedside. According to students, focussing more on practically oriented clinical skills with proper supervision would improve learning while hindering factors were large number of students and patient's uncooperativeness. Good communication was considered the best method of alleviating patient discomfort at bedside teaching in this study.

**Conclusions:**

The study concluded that medical students have positive response and learning attitudes towards different aspects of bedside teaching.

## INTRODUCTION

Bedside teaching involves 'presentation of new cases clerked by students, demonstration of physical signs, providing feedback to students, showing humane ways of sharing bad news and physician's personal interaction with patients'.^1^

Bedside teaching was a key learning modality over the last century which represented 75% of all forms of clinical training.^2^ However at present, in the modern era of medical education, it constitutes of less than 16% of the time spent on teaching rounds.^2^ Although a famous medical educationist said 'bedside teaching can only be a good thing',^3^ it is evident to seek contextual answers as to why there is a decline in bedside learning even in Nepal and to take opinion of one party of this learning process, the medical students.

The objectives of this study were to identify the perceptions of medical students towards different aspects of bedside learning, to identify barriers and to identify or establish factors that could improve learning at bedside at a medical school in Nepal.

## METHODS

This was a descriptive cross-sectional study done on third, fourth, final year medical students and interns at KIST Medical College, Lalitpur, Nepal from November to December 2014. A questionnaire was developed based on different aspects of bedside teaching using literature search, personal experience of authors and advice of experienced clinical educators. Questions related to medical students' perception towards bedside teaching, learned skills, confidence about patient examination and clinical management, factors that improve and hinder learning, group dynamics among students, perception about patients discomfort and their remedies learned during bedside teaching were included. This 34 item questionnaire was a combination of Likert scale, closed and open ended questions. These questionnaires were distributed to all the third, fourth, final year medical students and interns after information about the study. Those students who gave verbal consent and returned the filled questionnaire wereincluded in the analysis. Ethical approval was taken from the institutional review committee before the initiation of study. The answers of open ended questions were analyzed to create a theme and which was then coded. Data was entered and analyzed using SPSS 17.0. Frequency and proportions were calculated.

## RESULTS

Students who gave the consent to take part on the study and filled the questionnaire were included. About 306 out of 344 questionnaires were returned by the students and enrolled for analysis.

Almost all 304 (99.3%) of medical students considered that bedside learning is a useful learning tool in medicine and the duration of which should be around 143 min per session (SD±67 min with minimum of 25 and maximum of 420 min). Many of students 233 (76%) were satisfied with the existing method and process of bedside teaching employed for learning i.e. discussion of history taking followed by examination and management of patient. About 223 (73%) of students agreed with instructor demonstrating the clinical examination followed by students to elicit the sign employed at learning at bedside. Majority of participants 196 (64%) responded that everyone does not get equal or absolute opportunity to examine a patient individually for bedside learning. Majority felt confident about history taking and physical examination 251 (82%) and patient management 184 (60%) after bedside learning session.

About 150 (49%) of medical students agreed while 107 (35%) disagreed that discussion of case history in front of patient is appropriate during bedside teaching. However 227 (74%) thought that tutorial room is an appropriate site for the case discussion after patient history and physical examination. Most 239 (78%) medical students thought that theoretical lessons taught in classroom are applicable during bedside teaching.

Most of medical students considered bedside teaching provide active learning in real context 291 (95%). However 205 (67%) of total students felt that video demonstration of clinical signs and examination is an inferior method of learning than bedside learning on real patients. Similarly 234 (77%) of students preferred real patients over simulated patients in learning skills. Medical students also suggested various techniques to improve bedside teaching which are shown in (Table 1).

**Table 1.** Methods suggested by students to improve learning at bedside.

Response of students	Response percentage
Focussing more on practice oriented clinical skills with proper supervision/case discussion/practice	32.3%
Small group of students/sub-grouping of large group	24.2%
More number of patients /exposure to variety of cases	23.8%
Pre-developed bedside teaching schedule	14.9%
Active participations of students and teachers at bedside teaching	13.8%
Constant supervision/availability, punctuality of teachers at bedside	9.7%
Increase duration of bedside teaching/effective time management/more number of clinical class	7.4%
Motivation, punctuality, discipline and learning attitude of students	6.3%
Pre-informing patients, arranging cooperative patients and maintain patients comfort and privacy	5.9%
Suitable discussion place/space	4.5%
Clinical demonstration with audio-visual tools	2.2%
Inclusion of clinical posting in early years	0.4%

Key reasons in attending bedside classes were that bedside learning was interesting 257 (84%) and useful for learning 291 (95%). However 49 (16%) students attended these classes because of the mandatory attendance. Many medical students were not satisfied fully with their own interaction with patients, and patient's cooperation during bedside learning. Most 122 (40 %) disagreed and 104 (34%) agreed that all medical students in their group participates and interacts with each other during bedside sessions.

Medical students considered bedside teaching causes patient discomfort 184 (60%) and they suggested methods to pacify patients discomfort at bedside teaching (Table 2).

**Table 2.** Techniques suggested by students to decrease patient discomfort during bedside teaching.

Response of students	Response percentage
Good communications skills (reassurance, consent, counselling, empathy, rapport building)	59.1%
Maintaining privacy/confidentiality/professionalism	29.3%
Avoid repeated exam on same patient/less number of students examination on a patient	25%
Avoid discussion in front of patient/discussion in tutorial room	15.2%
Teachers' presence during session	3.6%
Prior skills and knowledge /theoretical aspects before bedside teaching session	2.8%
Simulated /clinical skills lab/audio-visual tools/video demonstration	0.4%

Medical students agreed that bedside learning has helped them to improve communication skills 294 (96%), doctor patient relationship 285 (93%), presentation skills 257 (84%), and to understand ethical and moral principles and patients feelings 263 (86%). Medical students 260 (85%) thought that bedside learning improved their overall clinical skills. Many medical students thought pre-developed schedule 141 (46 %) or based on availability of patients at bedside 144 (47%) should be method of case selection during bedside. The survey also highlighted factors that hinder or interfere during bedside learning which are depicted in (Table 3).

**Table 3.** Factors hindering learning at bedside.

Response of students	Response percentage
Large number of students	40.2%
Uncooperative patients/patients' visitor, relative and family uncooperativeness	26.6%
Less number of patients/cases/exposure	19.9%
Teaching attitude of teacher (specific learning attitude absent, no demonstration, no enthusiasm, no preparation, no supervision)	10%
Patients' discomfort (fear of causing discomfort, ill patient examination)	8.5%
Students' discomfort (Hunger, prolonged standing, backache)	7.4%
Lack of separate space for discussion	7%
Lack of students' self- study and preparation	5.9%
Inappropriate teaching environment with fear of teachers, scolding	4.8%
Less time duration of bedside teaching	4.1%
Lack of individual opportunity to examine at bedside	3.7%
Lack of availability of teachers	3%
Lack of students' discipline, motivation and punctuality	2.6%
Poor group dynamics among students	1.8%
Schedule related /specification of class hours	1.5%

The Cronbach's alfa was calculated to determine the internal consistency of questionnaire in 30 item Likert scale was 0.73.

## DISCUSSION

This study found that medical students had positive feelings about bedside teaching; in that they are useful method of learning in medicine and contribute to learn medicine actively in real context and also helps to understand ethical, moral values and patient's feelings. There was overall positive response towards different aspects of bedside teaching in this study. The students also provided feedback on various factors that are barriers to learning and suggestions for improvement in bedside teaching.

Historically, bedside clinical teaching began at Padua (Italy) and was formally established when the model of bedside teaching was developed by Herman Boerhaave (1688-1738) at Leyden (Netherlands). He made bedside clinical teaching an art, demonstrating and training most aspects of clinical practice.^4^ After a century, Sir William Osler (1849-1919) also emphasized: 'Medicine is learned by the bedside and not in the classroom'. Sir Osler was a key promoter of bedside teaching, who created a remarkable space for bedside teaching in modern day of medical education.^5^ In spite of evidence that medical students, teachers and patients are positive about bedside teaching improving clinical skills in medical students,^3,6^ the practice of bedside teaching is declining. The availability of sophisticated diagnostic modalities has improved early diagnostic accuracy so that less number of patient are admitted in ward, presume fear of breach of patients privacy, focused learning based on simulation techniques and availability of clinical skill labs are some of the reasons behind the decline in the use of bedside teaching with shifting of patient based learning to tutorial room learning.^3,6^ A busy physician who is unable to focus and contribute at bedside teaching due to overburden of work, limited time allocations for bedside teaching, negative perceptions towards bedside teaching due to fear of causing discomfort to patients are the other factors that impede bedside learning in the modern era.^3^

Bedside teaching brings three parties together i.e. the patient, medical student and clinical teacher for the process of learning. There is a constant dynamic interaction between these parties with sharing of information in-between them, ultimately benefiting everyone.^7^ The patients are central in the bedside learning in clinical medicine. Medical students during interactive sessions with patients at bedside not only develop skills of history taking but also understand ethical and moral principles, learn the different aspects of doctor-patient relationship, develop empathy and understand impact of illness on people which will guide them to develop professionalism in the future.^3^

The diagnostic medical technology, innovative audiovisual aids, clinical skills lab started dominating medicinal field and medical education over years in both developing and developed countries. However how sophisticated medical technologies can be, patient's empathy, sensitivity during history taking cannot be taught at classroom and skill labs.^1,3^ Bedside teaching in modern era should not only be limited to indoor patients but also has to be extended to outdoor settings, emergency, laboratory services, x-ray department and any other place where students can learn skills in the presence of patients.^8^ There should be formulation of new methods to teach clinical skills at bedside with collaboration between bedside teaching and technology.^9^ Therefore the technology should not be substitute to a bedside teaching but a supplement for better learning.

There are different 'models' proposed to make bedside teaching learning more effective, interesting and a learning experience for the students.^8,10^ There are different valuable suggestions and tips for successful bedside teachings by experienced medical educators.^8,11^ The most of these suggestions focus on active participation of students, facilitators and the patients. The medical students in this study also highlighted that active participation of students and teachers at bedside improves learning for better learning. The facilitator should be a role model to medical students at bedside in dealing with patients, showing an empathic response to patient's problems and able to integrate patient and medical student into management team. Clinical teacher acts as a bridge between patient and students making a conducive learning environment towards a holistic approach to patients' management at a bedside.^12^ The medical students in this study assumed that process of history taking and physical examination causes patient discomfort however study suggested that patients had positive attitudes towards bedside teaching and patients thought that medical students should examined patients during learning period at medical school.^13^ However the same study found that patients are satisfied and cooperative when examine by small number of medical students.^13^ Good communication skills, reassurances, professionalism, maintaining privacy and confidentiality were few important factors that were brought up in survey by medical students to help decrease patient discomfort during bedside learning in this study.

The most of the medical students (76%) were satisfied with process of one person taking history and performing physical examination while others assisting him/her in the process and eliciting signs individually afterwards. This was similar to technique followed in Australian medical school.^14^ However the mean duration of bedside teachings in our study was very high 143 minutes per session (2 hour 23 minutes per session) about 14 hours/week which is very high compared to study done among Australian medical students where it was 2-3 hour/week. The reason for significant difference in the duration of bedside teachings may be due to availability of clinical skills laboratory, simulation technique and advanced audiovisual demonstration techniques employed simultaneously with bedside teaching in Australian and other western medical schools while such facilities are lacking in Nepalese medical schools. The preferred site of discussion after history taking and physical examination was tutorial room and only few students supported the idea discussing in front of patients which was similar to previous studies.^7^

Bedside is not only a hub of learning clinical skills but also a place of nurturing communication skills, time management and record keeping.^7^ The bedside learning also helps to learn the practice of evidence based medicine and problem based learning.^7^

The students reported that large group of student, patient's uncooperativeness and lack of exposure to diverse clinical cases impair successful bedside teaching in this study which were similar to other studies around the globe signifying that perception of medical students around the globe is similar towards the bedside teachings.^15^ Medical students perceived that decreasing the student's number in a group with exposure to diverse cases, active participation of students /facilitator and patient would help to improve learning. The medical students assumed that increased exposure of students to a large number of patients during rotation to various departments improves learning but evidence suggests small positive correlation between number of patients and improved clinical performance.^16^

The study was done at a single medical school and generalization of its results to large number of medical students should be done with utmost caution. Perceptions about bedside learning may also be influenced by students' learning experience prior to this study. The authors recommend that more studies should be carried out in many medical schools to find out perceptions of medical students towards bedside teaching. It is also recommended to carry out research to find out novel methods of bedside learning so that it is pleasurable experience to students throughout medical career.

## CONCLUSIONS

The study concluded that almost all medical students perceive that bedside teaching is a useful learning method in medicine and there was overall positive response of medical students towards different aspects of bedside teaching. The study also highlighted that bedside learning could be made more effective with active participation of all the parties focussing on learning skills.

## References

[1] Asani M. (2014). Bedside teaching: An indispensable model of patient-centred teaching in undergraduate medical education. Niger J Basic Clin Sci..

[2] La Combe MA. (1997). On bedside teaching. Ann Intern Med..

[3] Qureshi Z. (2014). Back to the bedside: the role of bedside teaching in the modern era. Perspect Med Educ..

[4] (1978). Boerhaave's men at Leyden and after. Med Hist..

[5] Stone MJ. (1995). The wisdom of Sir William Osler. Am J Cardiol..

[6] Peters M, Ten Cate O. (2014). Bedside teaching in medical education: a literature review. Perspect Med Educ..

[7] Nair BR, Coughlan JL, Hensley MJ. (1997). Student and patient perspectives on bedside teaching. Med Educ..

[8] Salam A, Siraj HH, Mohamad N, Das S, Rabeya Y. (2011). Bedside teaching in undergraduate medical education: issues, strategies, and new models for better preparation of new generation doctors. Iran J Med Sci..

[9] Swamy M, Searle RF. (2012). Anatomy teaching with portable ultrasound to medical students. BMC Med Educ..

[10] Stickrath C, Aagaard E, Anderson M. (2013). MiPLAN: a learner-centered model for bedside teaching in today's academic medical centers. Acad Med..

[11] Abdool MA, Bradley D. (2013). Twelve tips to improve medical teaching rounds. Med Teach..

[12] Wenrich MD, Jackson MB, Ajam KS, Wolfhagen IH, Ramsey PG, Scherpbier AJ. (2011). Teachers as learners: the effect of bedside teaching on the clinical skills of clinician-teachers. Acad Med..

[13] Alawad AA, Younis FH. (2014). Patients' Attitude towards Undergraduate Medical Students at University Charity Teaching Hospital in Sudan. Int J Med (Dubai)..

[14] Indraratna PL, Greenup LC, Yang TX. (2013). Bedside Teaching in Australian Clinical Schools: A National Study. Journal of Biomedical Education.

[15] Ahmed AM. (2010). Bedside teaching at the Cinderella status. Options for promotion. Saudi Med J..

[16] Kim JY, Myung SJ. (2014). Could clinical experience during clerkship enhance students' clinical performance?. BMC Med Educ..

